# The Effect of Egg Embryonation on Field-Use of a Hookworm Benzimidazole-Sensitivity Egg Hatch Assay in Yunnan Province, People's Republic of China

**DOI:** 10.1371/journal.pntd.0001203

**Published:** 2011-06-28

**Authors:** Andrew C. Kotze, Peter Steinmann, Hui Zhou, Zun-Wei Du, Xiao-Nong Zhou

**Affiliations:** 1 CSIRO Livestock Industries, St. Lucia, Brisbane, Australia; 2 National Institute of Parasitic Diseases, Chinese Center for Disease Control and Prevention, Shanghai, People's Republic of China; 3 Department of Epidemiology and Public Health, Swiss Tropical and Public Health Institute, Basel, Switzerland; 4 University of Basel, Basel, Switzerland; 5 Helminthiasis Division, Yunnan Institute of Parasitic Diseases, Pu'er, People's Republic of China; Ministère de la Santé Publique et de la Lutte contre les Endémies, Niger

## Abstract

**Background:**

Current efforts to control human soil-transmitted helminths (STHs) involve the periodic mass administration of benzimidazole drugs to school aged children and other at- risk groups. Given that high levels of resistance to these drugs have developed in roundworms of livestock, there is a need to monitor drug efficacy in human STHs. The current study aimed to evaluate an *in vitro* egg hatch assay for measuring the sensitivity of human hookworms to benzimidazole drugs in an isolated field setting in southern Yunnan province, People's Republic of China.

**Methodology/Principal Findings:**

Egg hatch assays were performed with hookworm (*Necator americanus*) eggs extracted from 37 stool samples received from local school-aged children. The mean IC_50_ was 0.10 ug/ml thiabendazole (95% CIs: 0.09–0.12 ug/ml). Observation of the eggs immediately prior to assay set-up revealed that a small percentage had embryonated in some samples. Scoring of % embryonation of eggs prior to the assay allowed for corrections to be made to IC_50_, IC_95_ and IC_99_ values. Examination of the data with and without this correction revealed that the embryonation of a small number of eggs did not affect IC_50_ values, but did increase IC_95_ and IC_99_ values for some samples.

**Conclusions/Significance:**

This study has highlighted the impact of egg embryonation on the use of benzimidazole drug sensitivity assays for human hookworms in field settings. Given the greater flexibility required in human stool collection procedures compared to livestock studies, we suggest that embryonation of some eggs may be an unavoidable issue in some human studies. Hence, it needs to be measured and accounted for when analysing dose response data, particularly for generation of IC_95_ and IC_99_ values. The protocols used in this study and our suggested measures for accounting for egg embryonation should have widespread application in monitoring benzimidazole sensitivity at field sites worldwide.

## Author Summary

With the implementation of mass drug administration programmes for the control of human soil transmitted helminths there is a need to develop drug sensitivity monitoring tools to detect the emergence of resistance. The present study aimed to use an egg hatch assay to measure benzimidazole sensitivity in human hookworms in a field setting in Yunnan province, People's Republic of China, in order to assess whether the assay offered a practical means of monitoring drug sensitivity in human hookworms in such a location. The assay proved able to generate dose response data, which allowed for the drug sensitivity of the hookworms in the local children to be described; the mean IC_50_ was 0.10 ug/ml thiabendazole. The study also found that practical issues associated with stool collection procedures, specifically the embryonation of some eggs during the time elapsing between stool deposition and egg recovery, can have an impact on the drug sensitivity data. We suggest means for data analysis that overcome the impact of egg embryonation on drug dose response data, which should allow for the use of such assays at different field sites worldwide.

## Introduction

Periodic mass administration of the benzimidazole drugs albendazole or mebendazole to school-aged children and other at-risk groups is the mainstay of all current programmes to control soil transmitted helminths (STHs) in humans [1]. The massive scale and increasing frequency of anthelmintic treatment mean that it is essential to monitor drug-exposed worm populations to ensure that any drug resistance is detected should it emerge. Early detection is a prerequisite for the implementation of mitigation strategies such as drug rotation to ensure that the effectiveness of the few existing anthelmintic drugs is preserved for as long as possible.

The detection of anthelmintic drug resistance has been the subject of much attention in the livestock area [2], [3]. The faecal egg count reduction test (FECRT), in which faecal egg counts are conducted before and after drug treatment to detect any reduced drug efficacy indicative of drug resistance, is the most readily available test that can be adapted for the human field. It is currently being assessed by the WHO as a drug resistance monitoring tool. However, this test suffers from a lack of sensitivity, and its performance depends on pre-treatment egg counts (infection intensity) and possibly density dependent fecundity [4], [5].

*In vitro* phenotypic assays and molecular biology-based tests have been studied extensively in livestock nematodes. An egg hatch assay has been described for measuring resistance to benzimidazole drugs [2], [3], and may therefore be applicable for the detection of benzimidazole resistance in human hookworms, the only common STH to hatch outside the human body. Molecular tests monitoring changes in beta tubulin genotypes have also been proposed [3], [6]. However, even in certain well–studied livestock parasite species the relationship between benzimidazole drug efficacy and genotype is not fully understood [3], and the importance of beta tubulin SNPs in benzimidazole sensitivity in human STHs has not yet been demonstrated. Hence, there is a need to develop and utilise phenotypic tests which examine the direct effects of a drug on the free living stages of the human STH *in vitro* until sensitive molecular tests become available.

The use of egg hatch assays for measuring benzimidazole sensitivity in human STHs in field sites has been reported by several groups [7], [8]. Recently, Kotze et al. [9] described a standardised egg hatch assay for human hookworms in a 96-well plate format using a concentration gradient of thiabendazole embedded in agar. The present study aimed to test this assay format at a field site in the Peoples Republic of China (P.R. China), and to examine logistical and technical issues such as sample collection, handling and storage, egg isolation and test evaluation which may have an impact on the use of the assay in the field.

## Materials and Methods

### Ethical approval

The present study is an integral part of an on-going project for helminth infection surveillance among schoolchildren implemented by the Yunnan Institute of Parasitic Diseases in collaboration with educational authorities. It focuses on the epidemiology and control of intestinal helminth infections in this province in southwest P.R. China. The project has been approved by the Academic Board (Ethics Committee) of the National Institute of Parasitic Diseases, Chinese Center for Disease Control and Prevention in Shanghai, P.R. China. Informed consent to conduct the present study was given by local school authorities who informed the eligible students in the presence of the responsible personnel from the Yunnan Institute of Parasitic Disease. As the children board at the school during the week, and are under the responsibility of the school, this information session was not done in the presence of the parents/guardians. The students were informed about the study aims, procedures and potential risks and benefits, and were alerted to the possibility to withdraw from the study at any time without further obligation. Hence, assent to participate in the study was indicated by subsequent submission of a stool sample. By withdrawing, children did not forfeit their right to anthelminthic treatment at study completion. This procedure, with consent provided by school authorities and by the study subjects through choosing to participate, with no requirement for individual written informed consent, is in line with national and local standards for such studies which are based on diagnosis with no invasive procedures, and was approved by the ethics committee. A single standard dose of albendazole (400 mg) was provided free of charge to all study participants and their classmates after collection of stool samples.

### Study site and design, and faecal sampling

**Figure 1:**
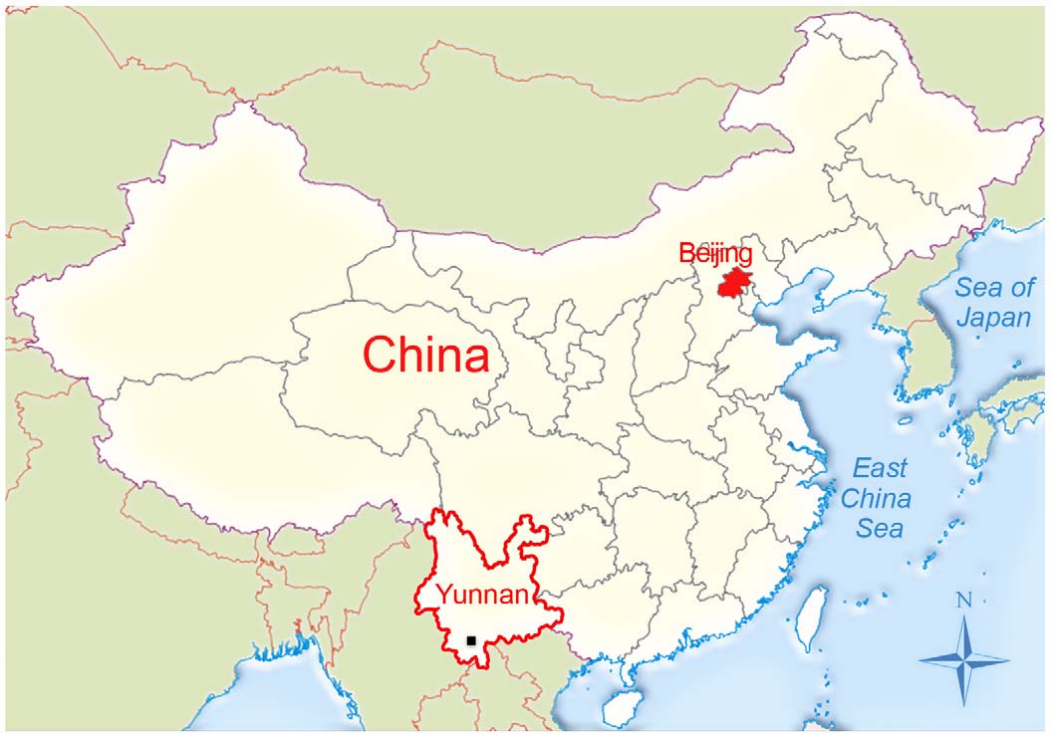
Location of the study site (▪) in Yunnan province, southwest P.R. China. The image was taken from a Public Domain Source.

Stool samples were collected among students of a primary school located in a suburb of Pu'er city, in southern Yunnan province, P.R. China (Figure 1). Labeled (identification number and name) stool collection containers were handed out to students in the afternoon, and students were advised to keep filled containers shaded and at room temperature. Filled containers were collected the next morning, and transferred to the local branch of the Yunnan Institute of Parasitic Diseases (YIPD), where all subsequent manipulations were performed, within 1 h. In the laboratory and during analyses, only the ID number was used to identify samples; a key linking ID numbers to names was retained by the YIPD.

### Egg recovery

Hookworm eggs were recovered from individual samples rather than pooling faecal samples in order to determine the range of responses in samples from within the same population. The presence of hookworm eggs in the collected samples was determined using the Kato-Katz method [10]. Whenever stool samples could not be processed for egg recovery on the day of collection, about 20 g of stool was suspended in ca. 40 ml water to arrest the development of hookworm eggs (anaerobic storage) [11], and the solution stored at room temperature.

The protocol for egg recovery was adapted from Kotze et al. [8]. About 20 g of the hookworm-positive stools, or whatever was available in case the sample was smaller, was mixed with water 1∶2, homogenized using a spatula and poured through a tea strainer. The runoff was transferred to 45 ml centrifuge tubes. The tubes were topped up with tap water, shaken carefully, and centrifuged in a bench top centrifuge for 2 minutes 30 seconds at 300 g. The supernatant was then poured off, the tube filled with saturated saline solution, and the sediment loosened using a wooden stick. Subsequently, the tube was centrifuged for 2 minutes 30 seconds at 130 g, and then left to stand for 5 minutes. The top 2 cm of the saline solution column were then transferred to a 15 ml tube using a disposable pipette. The 15 ml tube was filled with tap water and centrifuged for 2 minutes 30 seconds at 300 g, and the supernatant carefully removed using a disposable pipette. The tube was filled again with tap water, shaken and centrifuged as before. The supernatant was then removed leaving about 3 ml liquid above the sediment, and the sediment re-suspended.

Aliquots of 20 µl of the egg suspension were then placed on a slide and the number of hookworm eggs counted. Based on these counts, the egg concentration was adjusted to approximately 2 eggs per µl egg solution, or the highest possible concentration in cases where fewer eggs than required to achieve the target concentration had been recovered.

It was noted after the study had commenced that unusually high numbers of larvae were present in some drug assay plate wells with high drug concentrations. Subsequent observation of egg samples immediately following extraction from faeces revealed that some eggs had embryonated (embryos visible within the egg shell). Hence, from that time onwards the percentage of eggs that had embryonated prior to assay set-up was measured. A subsample of eggs was taken, and each egg was scored as being either embryonated (that is, with a larval shape visible within the egg) or not (that is, in a multicell stage). The mean number of eggs in the samples examined for embryonation (± SE) was 70±9. The number of samples for which embryonation was measured was 36. Only the egg hatch dose response data from these samples was analysed for the present study.

A PCR test designed to discriminate between *Necator americanus* and *Ancylostoma duodenale* was performed on a sub-sample of the recovered hookworm eggs conserved in ethanol and analysed according to the protocol published by Zhan et al. [12]. For DNA extraction, the DNEasy blood and tissue kit produced by Qiagen (Hilden, Germany) was used according to the manufacturer's protocol.

### Egg hatch assays

Assay plates were prepared as described by Kotze et al [9]. A stock solution of thiabendazole (10 mg/ml) was prepared in DMSO, and diluted 17.2-fold in DMSO to give a solution of 0.58 mg/ml, which was then serially-diluted 2-fold to produce a further 9 drug concentrations. Aliquots (2 µl) from this series of dilutions (starting at 0.58 mg/ml) were added to 96-well microtitre plates, such that each row of the plate comprised a gradient of ten dilutions. The first two wells of each row were utilized as control wells (i.e. received 2 µl of DMSO only), the 3^rd^ well contained the lowest drug concentration, and the 12^th^ well the highest. Each drug concentration was present in all 8 wells within each column of the plate. 200 µl of 2% agar (Davis Gelatine Co., powdered agar Grade J) was dispensed into each well of the plate and allowed to set. Thus, the concentration of thiabendazole across the plate ranged from 0.01 to 5 µg/ml (after addition of 30 µl of egg solution as described below). A piece of absorbent cloth soaked in an amphotericin B solution of 2.5 µg/ml was placed on top of the plate lids, and each plate was placed into a plastic press-seal bag and stored at 4°C. A box of plates was shipped at room temperature to the field site. The plates were refrigerated on arrival.

Aliquots of each egg solution (30 µl) were added to all wells in duplicate rows, or if insufficient egg solution was available, only every second well was loaded. The assay plates were incubated at 28°C for 48 hours before all larvae were killed by addition of 10 µl Lugol's iodine to each well. The number of larvae present in each well was then counted either by direct observation of the well under a dissecting microscope, or if too much debris or fungal/bacterial growth was present, the contents of the well was collected using a disposable pipette and transferred onto a microscope slide. In the latter case, both the empty well and the microscope slide were examined, and all larvae counted.

### Data analysis

As the use of IC_95_ and IC_99_ values has been advocated for livestock nematodes as more sensitive measures of changes in drug response in worm populations than IC_50_ values (for example, Coles et al. [3]), we examined the data in terms of IC_50_, IC_95_ and IC_99_ values. For each sample, the mean number of larvae present in the 4 control (no drug) wells was calculated. The number of larvae present in each drug well was then expressed as a percentage of the control mean. Data was analysed by non-linear regression using GraphPad Prism software in order to generate IC_50_, IC_95_ and IC_99_ values, representing the drug concentrations which inhibited egg hatch (reduced numbers of larvae present in wells) by 50%, 95% or 99% relative to control wells.

Dose response data were analysed before and after the application of a correction for the % embryonation which had been measured prior to assay set-up for each egg sample. This correction was done for each sample of eggs based on the % embryonation measured for that sample. Firstly, the number of eggs embryonated in each sample was expressed as a percentage. The mean number of eggs in the dose response assay control (no drug) wells was then corrected by removing the number of larvae that would be expected to have been derived from embryonated eggs added to those assay wells when the assay was established; for example if a mean of 80 eggs were present in control wells for a sample which had been shown to be 15% embryonated prior to assay set-up, then the control mean would become 80−(15/100×80) = 68 eggs. Similarly, the numbers of larvae present in each drug assay well was also corrected by subtraction of the number of larvae expected to have been derived from embryonated eggs in that specific egg sample ( = 12 in this example). The corrected drug assay data point was then expressed as a percentage of the corrected control mean.

Corrected and uncorrected dose response data were analysed in two ways: firstly, by pooling the % egg hatch values for all 36 samples at each drug concentration to generate a single dose response for the uncorrected and corrected data sets (and, hence single IC_50_, IC_95_ and IC_99_ values for each data set); and, secondly by generating separate dose responses (and hence, separate IC_50_ and IC_95_ values) for the data derived from each study subject's egg sample.

## Results

The PCRs designed to discriminate between *N. americanus* and *A. duodenale* indicated the presence of only the former species in the egg samples. This is in agreement with the known predominance of *N. americanus* in the study population (Steinman, unpublished data). Eggs extracted from 37 faecal samples were examined in egg hatch assays. One of these assays was not included in the subsequent analysis as less than 20 larvae were present in the control (no drug) wells of the assay plates at the end of the incubation period. Hence, a total of 36 separate assays were analysed by non-linear regression.

**Figure 2:**
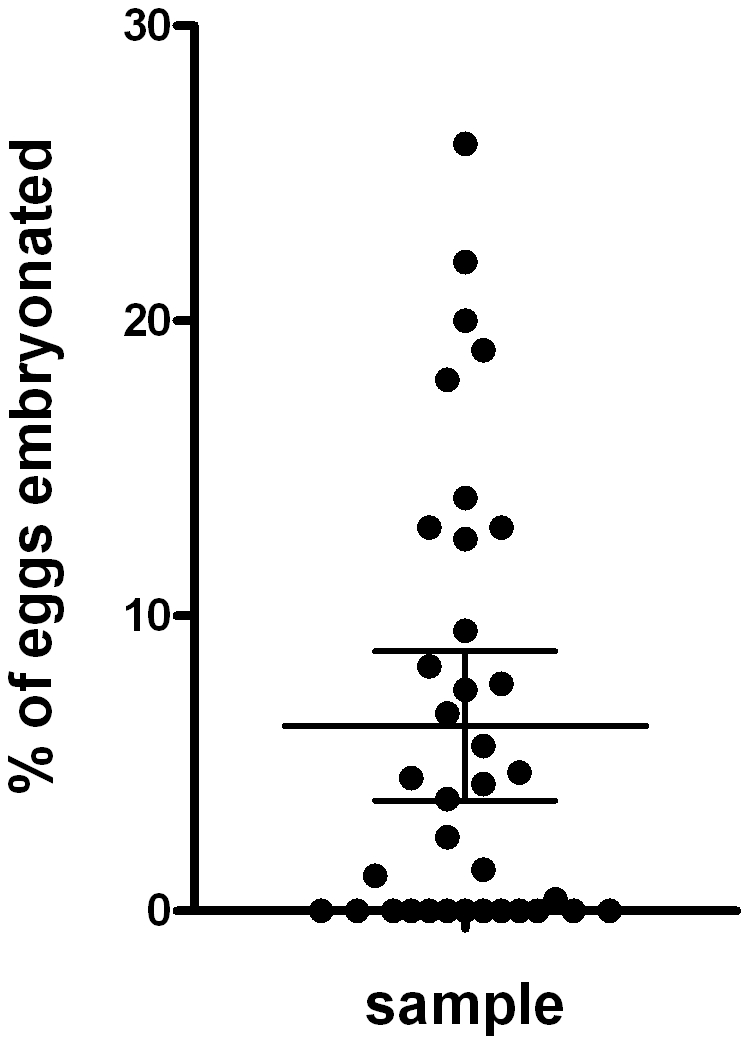
Percentage of embryonated eggs in samples used for egg hatch assays; solid line represents mean and 95% Confidence Intervals, n = 36.

The % embryonation measured in all samples prior to assay set-up is illustrated in Figure 2. The mean % embryonation was 6.3%, ranging up to 26% in one sample. The number of samples in which at least one egg had embryonated was 23 out of the 36.

**Figure 3:**
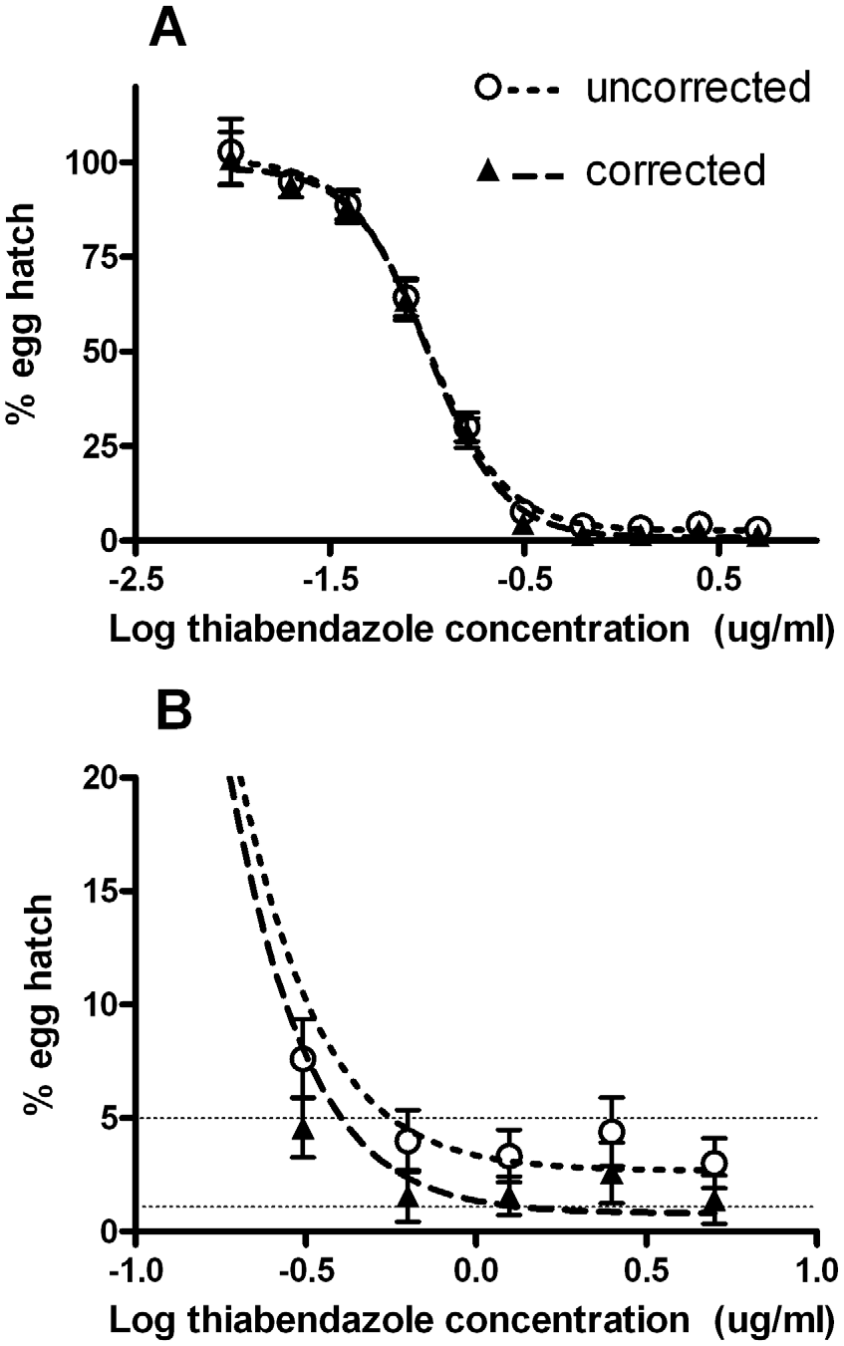
Dose responses shown by pooled egg hatch assay data from the embryonation uncorrected ( ○ ) or corrected ( ▴ ) data sets. **A**: full dose responses, **B**: portion of dose responses with egg hatch <20%. Horizontal dotted lines in **B** represent the IC_95_ and IC_99_ response levels.

Initially, we pooled the assay data to examine the overall population dose responses using data either corrected for embryonation or uncorrected (Figure 3, Table 1). Sigmoidal dose response relationships were apparent (Figure 3A), with the responses for both data sets showing very little difference at either the IC_50_ or IC_95_ levels, with overlapping 95% CIs (Table 1). Both data sets showed a plateauing of the response at the highest drug concentrations (Figure 3B). As a consequence, the uncorrected data set did not decrease to an IC_99_ level, while the embryonation-corrected data set did decrease to a level which allowed for the calculation of an IC_99_ (Figure 3B, Table 1). However, the egg hatch in this latter data set did not reach zero even at the highest thiabendazole concentration of 5 ug/ml.

**Table 1:** IC_50_, IC_95_, and IC_99_ values for pooled data either uncorrected or corrected for embryonation.

Data set	IC_50_ (95% CI)(ug/ml)	IC_95_ (95% CI)(ug/ml)	IC_99_ (95% CI)(ug/ml)
Uncorrected	0.105 (0.095–0.117)	0.583 (0.415–0.818)	>5
Corrected	0.103 (0.092–0.115)	0.407 (0.304–0.544)	1.60 (0.91–2.83)

**Figure 4:**
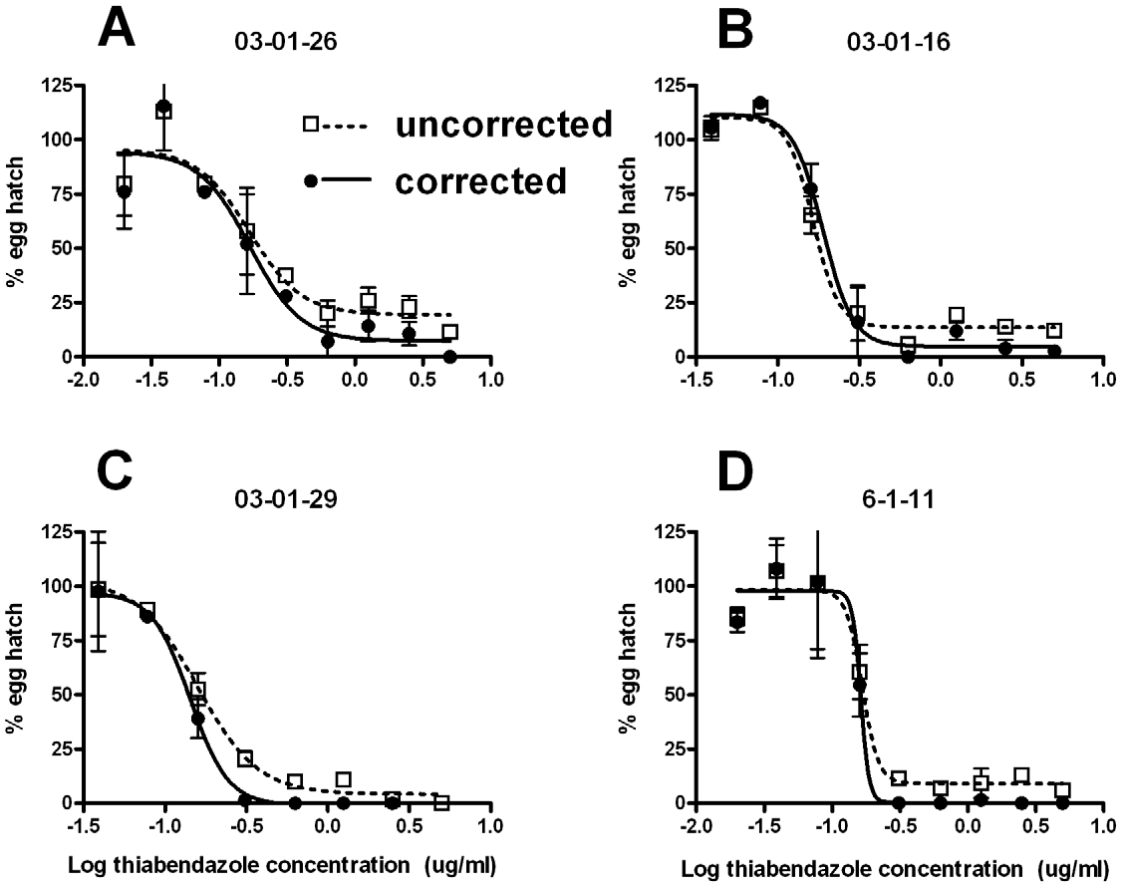
Dose responses for eggs recovered from 4 individual study subjects. Parts **A–D** show data from individual subjects (ID numbers shown above each graph) either uncorrected (□, dotted lines), or corrected (•, solid lines) for embryonation. Each data point represents mean ± SE, n = 2.

The relationship between egg hatch and drug concentration in egg samples recovered from individual subjects is illustrated with 4 examples in Figure 4. In each case a plateau was present in the uncorrected data sets, with the % egg hatch at levels of 5–25% at the highest drug concentrations. After correction for embryonation, two effects were apparent: in A and B, the level of the plateau was reduced, but a plateau was still present; in C and D, the plateau was removed, and egg hatch was reduced to zero at the highest concentrations. In all cases the curves showed little change with regard to the IC_50_ point following correction for embryonation.

**Figure 5:**
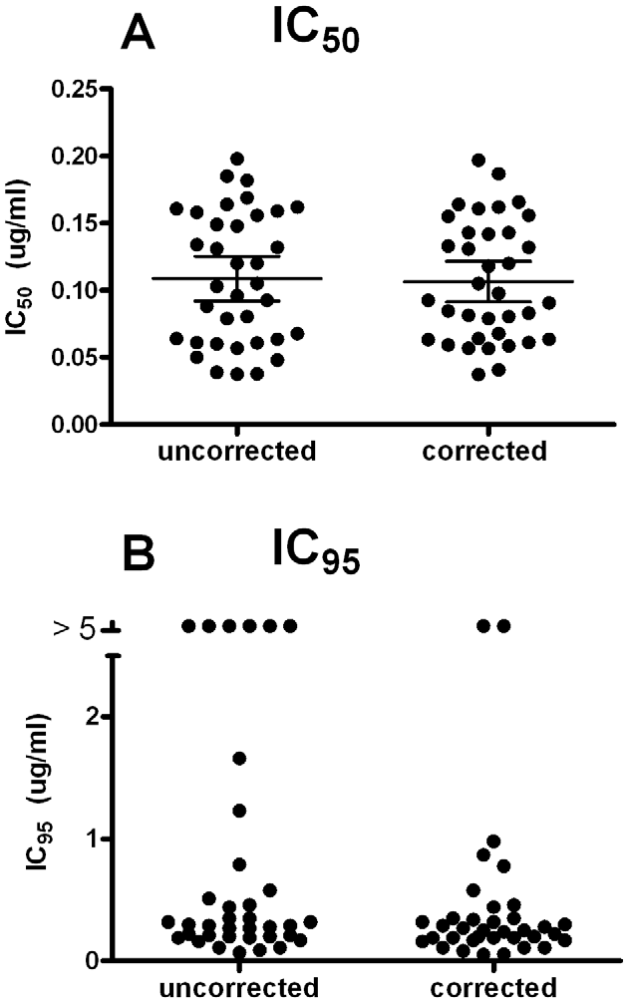
IC_50_ and IC_95_ values for egg samples from each study subject, from either embryonation uncorrected or corrected data sets. Parts **A** and **B** show IC_50_ and IC_95_ values, respectively. Horizontal lines represent mean and 95% Confidence Intervals.

The variation in IC_50_ and IC_95_ values among the different individual faecal samples is illustrated in Figure 5. IC_50_ values showed a similar range (approximately 5-fold) across both data sets, and mean IC_50_ values were not significantly different before and after embryonation correction (paired t-test, *P* = 0.33). A comparison of the IC_95_ values for the uncorrected and corrected subsets in Figure 5B illustrates the effect of applying the egg embryonation correction in reducing the number of samples for which an IC_95_ could not be calculated (that is, IC_95_>5 ug/ml). However, even after the correction an IC_95_ could not be calculated for two samples. These two outlying IC_95_ values were derived from samples showing embryonation rates of 14 and 8%.

## Discussion

The present study has generated baseline data for drug sensitivity of human hookworms (overwhelmingly *N. americanus*) in a field setting in southwest P.R. China, and raised a number of important issues if such assays are to be standardized for widespread use. The IC_50_ for the pooled data (0.10 ug/ml, 95% CIs 0.09–0.12) was similar to that reported previously for *N. americanus* in Papua New Guinea by Kotze et al [8] (0.076 ug/ml) and in Pemba Island, Zanzibar, by Albonico et al. [7] (0.079 ug/ml), indicating a degree of consistency in IC_50_ determinations by such assays in quite different field settings.

The embryonation of some eggs is an important issue that has not previously been reported. Egg hatch assays in the livestock sphere are performed on eggs isolated from fresh samples (<3 hours old at the commencement of the egg extraction procedures), or, if this is not possible, the faeces is mixed with water and sealed in tubes to generate anaerobic conditions until egg extraction can commence [2], [11]. Le Jambre [13] compared egg hatch assays using embryonated and unembryonated eggs of *Haemonchus contortus*. The IC_50_ was approximately 10–20 fold higher if embryonated eggs had been added to assays than if unembryonated eggs had been used. In the present study, a degree of embryonation was observed and measured in some samples. Significantly though, this did not affect the pooled data IC_50_ and IC_95_ values (from Figure 3 and Table 1), or the mean IC_50_ values derived from corrected and uncorrected data set from individual study subjects (Figure 5).

The embryonation of some eggs did, however, have a significant effect on some individual dose responses, in particular the IC_95_ and IC_99_ values for these samples (from Figures 4 and 5), as well as the IC_99_ for the pooled data (Figure 3B and Table 1). The presence of a small proportion of embryonated eggs manifested itself as a plateau in the dose response curves. This was most likely due to the shorter period of drug exposure prior to hatching for these eggs compared to the majority, hence allowing them to hatch at drug concentrations that would otherwise have been lethal. Although the levels of embryonation observed here (mean of 6.3%) did not affect the IC_50_ and IC_95_ for the pooled data (as described above), the illustration of the impact of the egg embryonation on individual cases in Figure 5, and its effect on IC_99_ (from Table 1), and the data described above from Le Jambre [13], indicate that it could potentially have significant effects on IC_50_, IC_95_ and IC_99_ values if it occurred at higher rates and / or in a greater proportion of individual samples than seen in the present study.

Hatching of eggs at high drug concentrations could either be indicative of the presence of a small proportion of worms able to resist the effects of the drug, or simply the presence of a degree of egg embryonation in the original sample. Hence, if embryonation was solely responsible for the dose response plateau then the correction we applied to the data may be expected to remove it. While this occurred in some cases (eg. Figure 4C and D), it was not achieved in others (Figure 4A and B), and also did not occur for the pooled data (from Figure 3B). This is likely due to experimental error rather than the presence of a real dose response plateau unrelated to embryonation. Our scoring of samples to obtain a % embryonation figure was based on a single sample of eggs for each faecal preparation (mean sample size ± SE = 70±9). Hence, this represents at best an estimate of the % embryonation in the samples. If this estimate was too low in a particular sample, its application to the data would not have removed the plateau in the dose response (as most likely occurred in Figure 4A and B). Hence, given the lessons learnt here, we suggest that a greater number of eggs are scored for embryonation at the time of assay set-up than was done for the present study. A sample size of at least 100 eggs may be suitable.

It would be clearly desirable to prevent embryonation prior to assay set-up when using egg hatch assays in field studies. However, this is not as easily achieved in human studies as in livestock surveys due to sampling constraints in the former. In the present study, faecal containers were handed out to children in the afternoon, and then collected the next morning. The faeces was then immediately analysed with the Kato-Katz method and, if found hookworm positive, covered with water and mixed well in order to prevent further egg development. Egg extraction then took place from approximately noon till mid-late evening. At this field site it would be difficult to reduce the time between stooling and the mixing of the samples with water in the laboratory since the local children do not stool very often, possibly associated with their low-fibre diet. Hence, it is not possible to only collect stools that had been deposited in the morning if a representative population sample is required. In other field settings, for example Kyrgyzstan [14], it would be quite easy to ensure the freshness of samples as containers can be given out in schools in the morning and then collected for processing 2–3 hours later. Hence, given this difference in stooling habits in different field settings, it may be difficult to standardise this aspect of the assay across all sites worldwide.

We advise that rather than try to standardise on a less desirable but more universal method that could be applied in all sites (that is, an overnight stooling period along with an acceptance of a degree of embryonation in some cases), every effort is made to ensure that samples are as fresh as possible, even if this means that different methods are applied at different field sites. That is, in areas where it is possible, containers should be distributed in the morning and collected again after 2–3 hours for processing. In other cases, an overnight stooling period would need to be accepted. Wherever freshness cannot be guaranteed, the % embryonation should be scored based on a sample of at least 100 eggs at the time of assay set-up, and all egg hatch scores corrected. In areas where fresh stool samples can be accessed, embryonation could be checked in some samples, but not necessarily in all. When stool freshness is assured, confidence can be placed in IC_50_, IC_95_ and IC_99_ values. Where stool freshness cannot be assured, and % embryonation corrections are required, IC_50_ values can be regarded with confidence, however, IC_95_ and IC_99_ values need to be judged carefully. Where high IC_95_ and IC_99_ values are observed, repeat samples could be collected.

There was a degree of variability between IC_50_ and IC_95_ values over the separate assays. The variability in IC_50_s from embryonation-corrected assays using different samples of worm eggs amounted to a 5.3-fold range. Kotze et al [8] found a 4.1-fold range in IC_50_ values among samples from individual subjects in a Papua New Guinean village. The range for the data of Albonico et al [7] was not reported, but the 95% CIs from that study were similar to those for the pooled data dose response in the present study.

The extraction of eggs from stools is a laborious process. In the present study, approximately 15 samples could be processed by 2 people working full-time in the laboratory every day, aided by another 2 technicians who performed the Kato-Katz test. The study of individual faecal samples allowed us to look at the variability across separate assays. However, such a procedure would not be necessary for a more general sensitivity-monitoring exercise. Hence, for such studies, a degree of pooling of faecal samples is recommended. We suggest that an approximately equal weight (or volume) of faeces from 5–10 individuals (depending on study size) could be pooled. Such a strategy has been applied previously by Albonico et al [7] who pooled faecal samples from groups of 10 children.

In conclusion, this study has once again shown that it is possible to measure drug sensitivity using an *in vitro* assay in a field site with limited laboratory equipment. Difficulties associated with the recovery of hookworm eggs (most significantly the freshness of the stools) are not prohibitive, but need to be accounted for during test performance and as part of the data analysis. The protocols used in this study, and our recommendations concerning the pooling of samples and the accounting for egg embryonation, should have widespread application. Although the ability of the egg hatch assay to detect benzimidazole resistance in human hookworms has not been proven (in the absence of known resistant populations), its utility for the detection of benzimidazole resistance in livestock parasites [2], [3] should generate a degree of confidence that it will also be applicable in the human sphere. There is therefore a need to apply these assays widely in order to obtain baseline data for drug sensitivity in different human hookworm populations so that changes associated with the emergence of drug resistance may be detected, and to compare drug-naïve populations with those already exposed to repeated drug treatments.
